# Dairy cows fed a low energy diet before dry-off show signs of hunger despite *ad libitum* access

**DOI:** 10.1038/s41598-019-51866-7

**Published:** 2019-11-06

**Authors:** Guilherme Amorim Franchi, Mette S. Herskin, Margit Bak Jensen

**Affiliations:** Aarhus University, Department of Animal Science, Blichers Allé 20, 8830 Tjele, Denmark

**Keywords:** Behavioural ecology, Behavioural ecology, Evolutionary ecology, Evolutionary ecology, Animal behaviour

## Abstract

Drying-off is one important management step in commercial dairy farms and consists of ceasing milk production artificially at a specific point in time, generally 2 months before the next calving. Drying-off typically comprises dietary changes as well as gradual or abrupt changes in daily milking frequency, which may challenge the welfare of high-yielding cows. This study investigated the isolated and combined effects of different feed energy densities (normal lactation diet versus energy-reduced diet, both offered *ad libitum*) and daily milking frequencies (twice versus once) on the feeding motivation of dairy cows on two separate days prior to dry-off (i.e. the day of last milking) using a push-gate feeder. During both days, cows on the energy-reduced diet pushed more than five times more weight to earn the final feed reward and were nearly ten times faster to feed on the first reward than cows on the normal lactation diet. Illustrating the importance of developing more animal welfare-friendly dry-off management, these results illustrate that cows show signs of hunger prior to dry-off when provided a diet with reduced energy density, although offered for *ad libitum* intake.

## Introduction

One important management step in dairy production worldwide is that milk production is artificially stopped at a specific time point during lactation, typically around 60 days before the next calving, in a process called drying-off^1^. The resulting dry period covers the last weeks of gestation. Drying-off abruptly is a common practice in many commercial dairy farms^2,3^, but dry-off management may also be gradual, including feeding low-energy diets and reducing the milking frequency prior to the day of last milking^2,4,5^. A dry period before the next lactation ensures regeneration of udder cells, increases milk production in the subsequent lactation and makes it easier to treat any intramammary infections^6,7^.

Nonetheless, drying-off may challenge the welfare of cows. For instance, prior to dry-off, cows are typically moved from their resident group to a dry-off pen, which may cause stress due to a new environment and the establishment of new dominance relationships^8,9^. Furthermore, the sudden cessation of milking in high-yielding cows differs considerably from the gradual decline in milk production seen in the natural setting^10^. Consequently, sudden cessation of milking may increase udder pressure due to milk accumulation in the udder and result in discomfort^11^, higher incidence of milk leakage and intramammary infections after dry-off ^8,12^. Concomitantly, cows may undergo quantitative or qualitative feed restriction to reduce nutrient supply to the udder and thus milk yield before the last milking^2,13^. To some extent, this may accustom cows to feed from dry cow rations that contain very low energy density. However, earlier studies reported metabolic stress^13,14^ and enhanced frequency of vocalisations in cows fed either a quantitatively restricted diet^8^ or an energy-reduced diet for *ad libitum* intake^15^ before the last milking, which suggests some deleterious consequences of these feeding strategies. In addition, continuing feeding a lactation diet until the last milking could benefit cows by avoiding a negative energy balance prior to the onset of the dry period, even though the negative shift in diet quality on the dry-off day would be more abrupt. However, only little is known about the effects of dry-off management on feeding motivation in dairy cows. One way to gain knowledge about specific consequences of drying-off on feeding motivation is to conduct a standardised behavioural test stimulating a meaningful behavioural response specifically related to the alleviation of a putative caloric insufficiency^16^.

Motivational tests can be based on operant responses and are then reliant on a trade-off between a desired resource and the performance of an operant response to gain access to the resource in question. Although no work to date has focused on cows around dry-off, different paradigms have been applied to investigate feeding motivation in cattle. For instance, requiring animals to walk long distances^17,18^, to push a weighted gate^19,20^ or to press a lever^21^. Irrespective of the required response, the methods are based on consumer demand theory^22,23^ and an individual’s motivation to pay a progressively increasing cost (i.e. to work harder and harder) to maintain access to a valued resource^24^. Furthermore, operant responses may be combined with other measures of resource use, such as latency to make an operant response^25^ and time spent with a resource once access is earned^26,27^.

This study aimed to investigate the isolated and combined effects of different feed energy densities (normal lactation diet versus energy-reduced diet, both fed for *ad libitum* intake) and daily milking frequencies (twice versus once) on the feeding motivation of dairy cows on two separate days prior to the last milking using a weighted push-gate feeder. We hypothesised that cows on the energy-reduced diet would be more motivated to feed and consequently display a shorter latency to earn the first feed reward and push progressively, increasing weight on the gated feeder in order to obtain successive feed rewards. Besides, it was expected that a concomitant twice-daily milking would augment the feeding motivation due to a larger nutrient requirement.

## Materials and Methods

This study took place from February to August 2018 at the Danish Cattle Research Centre, Aarhus University, Foulum, DK-8830, Tjele, Denmark, and was part of a larger experiment investigating the effects of different dry-off strategies on productivity, metabolism and welfare of high-yielding dairy cows. All procedures involving animals were approved by the Danish Animal Experiments Inspectorate in accordance with the Danish Ministry of Justice Act No. 1306 (November 23, 2007), approval number 2017-15-0201-01230. Information regarding the resident herd and housing, the larger experiment including composition of the diets, the animals in the larger experiment and illustrations of the experimental set-up can be found in the Supplementary Material.

### Experimental design, inclusion criteria and treatment allocation

As part of the larger experiment following a 2 × 2 factorial design with feed energy density (normal lactation diet versus energy-reduced diet, both fed for *ad libitum* intake) and daily milking frequency (twice versus once), cows were assigned to one of the four treatments from 7 days (D-7) prior to the day of the last milking (dry-off day).

All cows from the resident herd fulfilling the following criteria were enrolled in the study: lactating pregnant purebred Holstein cows; between 210 and 240 days of pregnancy on planned dry-off day; in their first to third lactation; yielding an average of minimum 15 kg milk per day 3 weeks prior to the last milking and with a body condition score of 2 to 4^28^ on D-14 relative to the dry-off day. Every two weeks, batches of 1 to 6 cows fulfilling these criteria were enrolled in the study and moved to an experimental pen on D-7. Cows previously enrolled in the experiment, cows with a locomotion score of 4 or 5^29^ within 2 months of the start of the experiment, or treated with antibiotics for clinical diseases between D-21 and D-14 relative to dry-off, were not enrolled in the study.

In total, one main experimenter (the first author) and seven assistants handled the cows and collected the data. Throughout the experimental period, experimenters were blinded to treatment allocation. Additionally, experimenters were blinded to daily milking frequency and partly blinded to the diets because these were visible in the feed bins. Within parity (primiparous and multiparous cows), cows were allocated to blocks of eight based on date of last calving and then allocated randomly to treatment within parity and block. Cows were walked by the barn staff to an automatic milking system (AMS) (DeLaval, Tumba, Sweden) to be milked either once (between 0530 h and 0700 h) or twice (between 0530 h and 0700 h, and between 1530 h and 1630h) daily. Fresh feed was delivered four times daily starting at 0630 h, 1030 h, 1400 h and 1930h, and the computerised feed bins (Insentec B.V., Marknesse, The Netherlands) were remotely locked for approximately 40 minutes while feed was delivered. The normal lactation diet was a partial mixed ration (PMR), and the energy-reduced diet was based on the same PMR but contained 70% of the normal lactation ration and 30% chopped barley straw (approximately 2 cm long) along with extra minerals in order to achieve the same mineral supply as the normal lactation diet^30^. Details of the two diets are shown in Table 1 in the Supplementary Material. In addition, each cow was offered a limited amount of concentrate (main composition described in the Supplementary Material) daily in the AMS. Cows on normal lactation diet were offered 3 kg of concentrate per day, while cows on energy-reduced diet were offered 1 kg of concentrate per day. The 3 kg was what cows were offered with the normal diet^31^. The 1 kg was to reduce concentrate allocation equivalent to the reduced PMR while still attracting the cows to the AMS^32^. From the dry-off day until calving and the onset of the next lactation, cows were fed a dry cow ration and were no longer milked.

### Animals included in the current study

Thirty-two [13 primiparous and 19 multiparous (mean ± s.d. = 1.7 ± 0.7)] lactating pregnant Holstein cows were included in this study. Their inclusion continued until eight cows per treatment were achieved according to a balanced two-sided one-way analysis of variance power calculation for 80% of power, medium-high effect size at 5% of significance level^33^. The tested cows (mean ± s.d.) weighed 765 ± 79 kg, were 231 ± 6 days in milk and yielded 25.4 ± 7.6 kg of milk per day on D-7.

Average daily energy intake was estimated for each treatment between D-6 and D-1. Calculations were based on net energy for lactation per kg of dry matter in each diet (Table 1 in the Supplementary Material) and the daily total dry matter intake (daily intake of the particular diet registered by the computerised feed bins and recorded concentrate intake in the AMS). Cows on the normal lactation diet and twice daily milking consumed (mean ± s.d.) 122.3 ± 29.0 MJ/d, cows on normal lactation diet and once daily milking consumed 128.8 ± 12.1 MJ/d, cows on the energy-reduced diet and twice daily milking consumed 66.6 ± 20.0 MJ/d and cows on the energy-reduced diet and once daily milking consumed 74.0 ± 19.4MJ/d. In summary, cows fed normal lactation diet consumed 126.0 ± 22.3 MJ/d against 69.4 ± 20.0 MJ/d consumed by cows fed energy-reduced diet, representing a difference of approximately 45% in daily energy intake.

In addition, average milk yield per treatment between D-6 and D-1 was estimated based on information collected by the AMS. Cows on the normal lactation diet and twice daily milking yielded (mean ± s.d.) 22.1 ± 9.2 kg/d, cows on normal lactation diet and once daily milking yielded 18.9 ± 6.3 kg/d, cows on the energy-reduced diet and twice daily milking yielded 18.8 ± 6.5 kg/d and cows on the energy-reduced diet and once daily milking yielded 15.2 ± 3 kg/d. Overall, cows milked twice daily yielded 20.4 ± 7.9 kg/d against 17 ± 5.2 kg/d yielded by once-daily milked cows, representing a difference of approximately 17% in daily milk yield.

### Experimental site and apparatus

The feeding motivation tests were conducted in a galvanised-steel-plated experimental hall (Future Rundbuehaller™, Tarm, Denmark) with natural ventilation (described in Supplementary Figs 1 and 2) located in the yard next to the resident barn at the Danish Cattle Research Centre, approximately 100 m from the home pen of the experimental cows. The hall was 12 × 10 m and had a ground flooring covered with 10 cm of sand. Two test pens and one centrally placed companion pen (each 5 × 3 m) were constructed from pen sides made of galvanised tubular bars.

In order to test the feeding motivation of the cows, two push-gate feeders (illustrated in Supplementary Fig. 3) were installed opposite to the entrance of each test pen. The gate of each feeder was 127 × 55 cm (height x width), weighed approximately 8 kg and was constructed from 3-cm-diameter tubular metal bars spaced 10.5 cm apart. The gates were equipped with a 72.5 × 50.0 cm (height × width) wooden board, creating a solid surface where the cows could push by use of muzzle or forehead. Attached perpendicularly to the outside of each gate were two 33 × 1 cm (length x width) metal bars on which up to 17 10-kg iron plates could be placed. Cows could see into a 47 × 27-cm trough, placed underneath the gate of the feeder and outside the pen, through a 7-cm bottom gap underneath the wooden board.

The dynamic force directed at the bottom and midpoint of the wooden board required to move the gate, without additional weight until it reached a hook and was held permanently open, was measured with a dynamometer (Sauter FK 500, Sauter GmbH, Balingen, Germany). In total, 10 measures were taken (five on the bottom and five on the middle point, the two points were 30 cm apart). The mean force required to open each of the four unweighted gates was included in the statistical analysis to control for differences in resistance between feeder gates. Across feeders, the mean force required to push the gates open were (mean ± s.d.) 28.8 ± 1.2 N and 44 ± 1.4 N for bottom and middle point, respectively. Regarding both points on each gate and all gates, the mean force required to push gates open was 36.4 ± 1.2 N. Additionally, the dynamic force directed at the bottom of the wooden board required to move the gate until it reached a hook and was held permanently open at every possible price (8 to 178 kg) was also measured. In total, two measures were taken at each possible price to calculate the mean force required to open each of the four gates. Across feeders, price and respective mean force to open each gate correlated almost perfectly (*r* = 0.99, P < 0.001).

### Procedures, training and testing

On the first day of the larger experiment (D-7), the barn staff separated the experimental cows from the resident groups between 0500 h and 0830 h to be milked before being moved to the experimental pen in the resident barn (as part of the larger experiment). During the separation, cows could feed as usual, while, in the experimental pen, feed bins were empty. Once the cows were in the experimental pen they had 30 minutes to familiarise with the new environment, after which they were clinically examined and marked for the purpose of the larger experiment. Subsequently, at around 1000 h, and before the experimental diets were delivered to the cows, they were walked in pairs or trios to the experimental hall for familiarisation to the test pens and trained to open the gates of the feeders. Thus, cows were feed-deprived for approximately 90 minutes before entering the experimental hall.

Upon arrival in the hall, the cow-pen allocation was first-enter-first-stay, and each cow was returned to the same pen on subsequent test days. In case of trios, one cow started in the companion pen and changed pen position with the first trained or tested cow. On the training day, each cow had 10 minutes to explore the test pen with the feeders open and empty. Subsequently, each cow went through a 3-step training process to learn to open the gate of the feeder that would be allocated with concentrate. One feeder, pre-determined randomly according to the cow-treatment allocation, was allocated concentrate (0.8 kg), and the gate was open. The first step the cows needed to fulfil was to feed on the concentrate for 3 seconds. If successful, the experimenter closed the gate and initiated the second step, where the cows needed to repeat step 1, but with the gate initially closed. First, the experimenter opened the gate, and slowly (holding the external metal bars) left a 5-cm gap between the gate and the trough. If the cow succeeded to push the gate until it reached the hook, which held the gate fully open, and to feed, the cow advanced to the third and final step, which was a repetition of step 2. After being trained with the concentrate, cows were offered uncut barley straw in the second feeder for 5 minutes. On D-5 and D-2 relative to the last milking (test days), cows encountered concentrate in the same feeder as during training. During the test days, uncut barley straw was available in the other feeder, which was open throughout the test (cost-free feed option). Five cows did not fulfil the learning criteria on D-7 and went through a repeated training process on D-5 before testing began. The average training time per cow, including the 5 minutes with access to straw, was 21 minutes. Two cows (both milked twice daily, but one fed the normal lactation diet and the other fed the energy-reduced diet) did not direct any attention to the concentrate and failed to reach the learning criterion on both days. Hence, they were not tested any further. Thus, 34 cows visited the experimental hall and 32 cows (from 10 batches) were included in the study.

On D-5 and D-2 between 1200 h and 1430 h, cows were calmly walked to the experimental hall following the same management and cow-pen allocation as during training. When the cows entered the test pens, the feeders were empty, closed and locked. Each cow was tested individually. First, the feeder that contained concentrate during training was allocated 0.8 kg of concentrate, while the other feeder was filled with uncut barley straw. Subsequently, the concentrate feeder was unlocked, but remained closed, and the straw feeder was unlocked and opened (and left open throughout testing). Cows then had 5 minutes to open the gate to the concentrate feeder and to feed on the concentrate (supplementary video). One outcome measure was the latency to feed concentrate for the first time. Every time a cow had fed on the concentrate for 3 seconds, counted from the moment the gate reached the hook and was held open, the experimenter closed and locked the gate. Then, the remaining concentrate was removed, a new 0.8-kg portion of concentrate was placed in the feeder and a 10-kg iron plate added onto the feeder within 30 seconds. This step was repeated (new 0.8-kg portion of concentrate and additional 10 kg on the gate) until 5 minutes had elapsed without any attempt to open the gate, or the cow reached the maximum weight limit of 178 kg (8-kg gate +17 10-kg iron plates). The final weight (including the 8-kg gate) pushed by each cow was noted as the maximum price paid to feed by the cow. The intake during each reward was recorded as 0.8 kg minus leftovers. The 3-second criterion was set to diminish the influence of individual differences in eating (e.g. bite size, eating rate). The amount of barley straw consumed was also recorded as initial amount provided minus leftovers. The test sessions lasted (mean ± s.d.) 13 ± 8 minutes. Moreover, the choice of test days was made to fit the weekly schedule of the larger experiment and to give cows the opportunity to behaviourally adapt to their respective feed rations.

### Variables and statistical treatment

Data were analysed in R version 3.6.1^34^. P-values < 0.05 were considered significant, values of 0.1 > P ≥ 0.05 were considered tendencies. The assessment of feeding motivation was based on the maximum price paid (weight pushed) to obtain a feed reward, latency to feed the first feed reward and eating rate (i.e. amount of concentrate consumed per 3-second visit). Each of these response variables was analysed using mixed-effects modelling. The initial models included feed energy density (normal lactation; energy-reduced), daily milking frequency (twice; once), test day in relation to the last milking (D-5; D-2), parity (primiparous; multiparous) and their interactions (2-way; 3-way; 4-way) as fixed effects. Cow and mean force required to open the particular unweighted gate were included as random effects. For each response variable, all non-significant interactions were removed sequentially using a backwards stepwise procedure with P > 0.2 as exclusion criterion, except the interaction between feed energy density and daily milking frequency. All 32 cows trained prior to testing were accounted for the analyses.

The linear mixed-effects model^35^ for maximum price included feed energy density, daily milking frequency, test day, parity and the 2-way interaction between feed energy density and daily milking frequency as fixed effects. Cow and force were included as random effects. The residuals were checked graphically for normality and homoscedasticity using Q-Q plot, histogram and plot of the residuals against the fitted values. No deviations from these two assumptions were found. Subsequently, post-hoc analyses were performed with Tukey-adjusted Least-Squares Means^36^.

The latencies to feed the first feed reward were assessed by Cox’s proportional hazards mixed-effects model^37^ using survival analysis^38^. The model included feed energy density, daily milking frequency, test day, parity and the 2-way interaction between feed energy density and daily milking frequency as fixed effects, and cow and force as random effects. The fit of the model was checked by assessing the significance of integrated log-link test. A hazard rate ratio (HRR) > 1 indicates a higher likelihood of feeding on one level compared with the other level of each categorical explanatory variable; meanwhile, 0 < HRR < 1 indicates a lower likelihood of feeding compared with the other level.

Additionally, eating rate was analysed using a linear mixed-effects model^35^. The model included feed energy density, daily milking frequency, test day, parity and the 2-way interaction between feed energy density and daily milking frequency as fixed effects, while the random effects were cow and force. The residuals were checked graphically for normality and homoscedasticity using Q-Q plot, histogram and plot of the residuals against the fitted values. No deviations from these two assumptions were found. Subsequently, post-hoc analyses were performed with Tukey-adjusted Least-Squares Means^36^.

Additionally, the proportion of cows that consumed barley straw in each treatment on each test day was analysed by Fisher’s Exact Test^34^.

## Results

The results are presented as least squares means and standard error of the mean (l.s.m. ± s.e.m.), unless otherwise stated.

**Figure 1:**
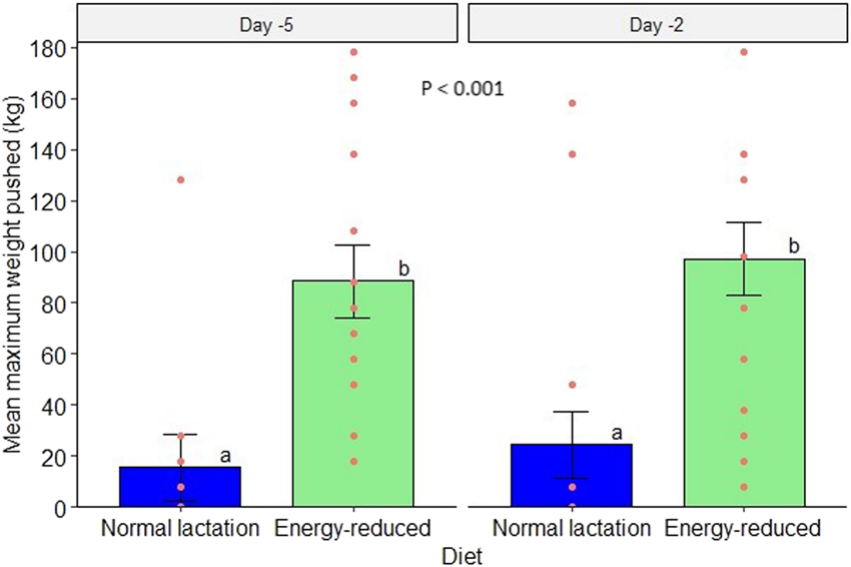
Mean maximum weight pushed to feed from the push-gate feeder. On both test days, cows on the energy-reduced diet (n = 16) were more motivated to feed and willing to work harder to earn feed rewards than cows fed on the normal lactation diet (n = 16). The bars represent the least-squares means, and the error bars indicate standard error of the mean. Different letters and bar fillings represent statistical differences at a significance level of P < 0.001 (two-tailed). The red dots represent each cow within each diet and illustrate the individual variation in maximum weight pushed.

**Figure 2:**
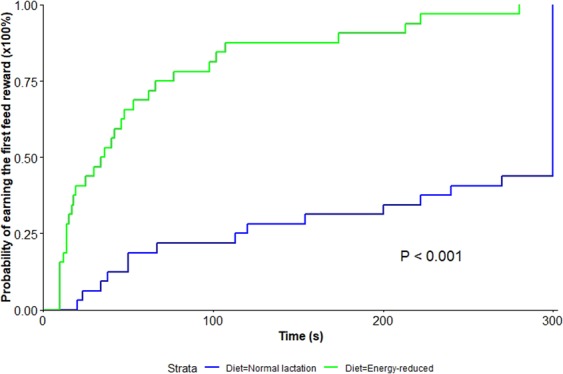
Survival plot of the probability of cows to acquire the first feed reward within 5 min. The Y-axis displays the probability of cows to approach a particular feeder and obtain the first feed reward, and the X-axis displays the time, in seconds, taken by the cows to start feeding. Over both test days, cows on the energy-reduced diet (n = 16) (green line) obtained the first feed reward much quicker than cows fed on normal lactation diet (n = 16) (blue line) prior to dry-off. Furthermore, less than 50% of the cows fed on the normal lactation diet earned the first feed reward within 5 minutes. Different line colours represent statistical differences at a significance level of P < 0.001 (two-tailed).

On D-5, eight cows did not engage with the feeder (all were fed the normal lactation diet, three milked once daily and five milked twice daily). On D-2, 10 cows (of which seven were the same cows as D-5) did not engage with the feeder (all were fed the normal lactation diet, three milked once daily and seven milked twice daily). Thus, these observations had zero maximum price, and latency was censored because the cows did not feed the first feed reward within the duration of the test (5 minutes). Cows on the energy-reduced diet displayed an increased motivation to feed as indicated by the higher maximum weight pushed to obtain feed rewards (Fig. 1) and by the shorter latency to earn the first feed reward compared to cows on the normal lactation diet (Fig. 2). Over both test days, cows on the energy-reduced diet reached a higher maximum price paid of 92.8 ± 13.4 kg compared to 19.9 ± 12.4 kg for cows on the normal lactation diet (F_1,26.7_ = 18.2, P < 0.001). Four cows (one on both test days and three on D-2), all receiving the energy-reduced diet, reached the maximum work limit. No significant effects of daily milking frequency (F_1,26.9_ = 0.6, P = 0.46), test day (F_1,31_ = 1.1, P = 0.31), parity (F_1,26.9_ = 0.3, P = 0.59) nor interaction between feed energy density and daily milking frequency (F_1,25.5_ = 0.7, P = 0.4) on the maximum price were found.

Cows receiving the energy-reduced diet took much shorter (median; IQR) (35 s, 14 to 69 s) to approach the experimental feeder and feed on the first feed reward than cows fed on the normal lactation diet (300 s; 118 to 300 s) (HRR = 8.5, 95% CI: 7.6 to 9.4, Wald (z) = 4.77, P < 0.001). No significant effects of daily milking frequency (Wald (z) = −0.9, P = 0.36), test day (Wald (z) = −1.2, P = 0.25), parity (Wald (z) = 0.6, P = 0.55) nor interaction between feed energy density and daily milking frequency (Wald (z) = 0.1, P = 0.93) on the latency to feed were found.

On both days, the concentrate consumption per cow ranged from 0 to 3 kg. The average amount of concentrate consumed per 3-second visit was 0.1 ± 0.01 kg. No significant effects of feed energy density (F_1,20.6_ = 0.4, P = 0.53), daily milking frequency (F_1,18_ = 1.5, P = 0.23), test day (F_1,20.7_ = 1.3, P = 0.26) nor interaction between feed energy density and daily milking frequency (F_1,19.6_ = 1, P = 0.32) on the amount of concentrate consumed per visit were found. However, primiparous cows consumed significantly more concentrate per visit (0.12 ± 0.02 kg) than multiparous cows (0.07 ± 0.02 kg) (F_1,18_ = 5, P = 0.037).

On D-5, 12 cows consumed barley straw [three cows on normal lactation diet and once-daily milking (0.03 ± 0.01 kg); two cows on normal lactation diet and twice-daily milking (0.04 ± 0.02 kg); four cows on energy-reduced diet and once-daily milking (0.13 ± 0.1 kg); three cows on energy-reduced diet and twice-daily milking (0.06 ± 0.05 kg)]. On D-2, 12 cows (half were the same cows as D-5) consumed barley straw [four cows on normal lactation diet and once-daily milking (0.05 ± 0.03 kg); two cows on normal lactation diet and twice-daily milking (0.16 ± 0.14 kg); three cows on energy-reduced diet and once-daily milking (0.02 ± 0.01 kg); three cows on energy-reduced diet and twice-daily milking (0.09 ± 0.06 kg)]. On both test days, no difference between treatments in the proportion of cows feeding on straw was found (odds ratio = 1.1, P > 0.1).

## Discussion

This study assessed how different dry-off management affects feeding motivation in cows using an operant feeder with a weighted push-gate where required weight pushed to acquire a fixed reward increased progressively. We confirmed the hypothesis that a higher proportion of straw in the normal lactation diet (reducing its energy density and consequently reducing the feed and energy intake) increases feeding motivation.

The typical practice of reducing nutrient provision to downsize milk synthesis prior to dry-off^2,4,13,14^ has been reported to have negative consequences for the welfare of high-yielding dairy cows. For instance, in studies where cows were fed only straw, they displayed deeper metabolic stress and higher plasma concentration of cortisol than cows receiving a silage-based diet a week before dry-off^4,13^. Similarly, cows fed only hay displayed relevant metabolic impairment compared to cows fed a lactation diet for 5 days prior to dry-off^14,39^. Moreover, an increased number of vocalisations in dairy cows fed a restricted amount of feed, or fed an energy-restricted diet, before dry-off have been reported^8,15^. While the higher cortisol levels may suggest physiological stress, the increased vocalisation may illustrate a behavioural stress response and negative emotional experiences^40^. However, both are general responses, and the current study is the first to investigate the feeding motivation in cows on an energy-reduced diet prior to dry-off by use of an operant-conditioning methodology. This paradigm allows an objective interpretation of specific behaviours (i.e. latency to earn the first reward, which herein reflects the motivation to obtain the concentrate and pushing a weighted door to access feed rewards) motivated by hunger^41^.

Unexpectedly, this study did not find any substantial effect of daily milking frequency on feeding motivation, which has been reported in other research^8^. We expected that milking cows fed an energy-reduced diet twice daily would increase their feeding motivation even more due to a higher milk yield and a resulting higher energy demand. However, the current results do not support the hypothesis that feed energy density and daily milking frequency simultaneously affect feeding motivation. This may be because diet had a greater effect on milk yield than milking frequency, but analyses of data from the larger study are necessary to clarify this. We tested cows after the first daily milking, approximately 5 hours after all experimental cows had been milked. Had the test taken place after the second daily milking, cows milked once daily might have been experiencing udder soreness^42^ due to milk having accumulated in the udder, which could potentially reduce their motivation to move and to push the gates.

We found no difference in feeding motivation between D-5 and D-2 relative to the last milking, and our results suggest that the feeding motivation did not change over days. Daily testing of feeding motivation from the initiation of treatments on D-7 could have enabled us to quantify the feeding motivation over time in more detail, and to assess more closely whether feeding motivation increases, or whether cows adapt to the energy restriction over days. However, in the present study, only a 7-day window was available for both training and testing, and our cows could only be tested twice with a 2-day interval between tests.

No effect of parity on the feeding motivation was detected in the current study. Primiparous cows are not physically mature and, as a result, may require extra energy for growth besides milking and maintenance^43,44^. Therefore, it could be hypothesised that they are more affected by decreased energy supply than multiparous cows. Nevertheless, our current results could not confirm this. Yet, primiparous cows consumed more concentrate per 3-second visit than multiparous cows. Generally, primiparous cows seemed more active when feeding, which made it more difficult for the experimenter to close and lock the gates within 3 seconds. Thus, primiparous cows may have fed for slightly longer than 3 seconds, which may also explain this result.

A secondary, however important, result of the present study is that dairy cows can be trained to perform an operant task within a relatively short time span. Only two of 34 cows failed to learn the task, and the remaining cows reached the learning criterion within 1 to 2 sessions. This success may be linked to a number of choices in the experimental plan. Firstly, an increased motivation to feed was achieved in all cows by feed depriving them for approximately 90 minutes prior to the initial training. In addition, the large board on the gate, that cows should push, allowed for slightly different ways of pushing due to variations in body size. Third, moving the cows in groups and testing them with visual contact to pen mates avoided the challenge of social isolation in a novel environment that otherwise could have compromised the performance of the tasks^45^. Fourth, the inclusion of a hook in each push-gate feeder, which made the price more uniform and permitted cows to eat freely after the price had been paid. Fifth, the choice of concentrate as a feed reward known to be highly palatable and energetic. Importantly, access to concentrate in the home environment was controlled and thus ensuring a closed economy. When the resource tested is freely available outside the test situation, there is a risk that this affects the measures of motivation because animals can reschedule resource use to after the test, and thus the pre-requisites of a closed economy are jeopardised^24^.

Besides the aforementioned reasons, we chose concentrate because we could not offer other highly energetic feed options without disturbing the experimental setup. Our experimental cows were lactating and accustomed to feed from concentrate in the AMS. Further, the amounts of concentrate consumed during testing were within reported ranges of daily concentrate consumption^31,46^. However, we acknowledge that the use of concentrate as a feed reward could impose limitations to tests of feeding motivation in cows in other parts of the production cycle. For example, dry cows are recommended to feed from lower energy diets to limit excessive weight gain and negative carryover effects after calving, such as negative energy balance and impaired reproductive performance^47,48^. Hence, if the same methodology was applied to assess the feeding motivation of dry cows, especially if tests were done on consecutive days, it would be imperative to reduce the amount of concentrate per reward and hence limit the maximum amount of concentrate one cow was allowed to consume. Finally, one could argue that the use of an attractive feed as reward could overestimate the feeding motivation of the cows, as the sight and smell of the resource per se may increase feeding motivation^41^. Nonetheless, the experimental cows from each diet treatment reached very distinct levels of maximum price, and it is unlikely that the results are due to the chosen feed reward.

The ceiling of the maximum weight may have underestimated the level of feeding motivation that some cows were going through. Previous studies found that dairy heifers offered restricted or low-energy diets pushed a range of 4.5^19^ to 63%^20^ of body weight on a weighted gate to get access to feed rewards. Even though those heifers were tested in different set-ups (i.e. push-gate apparatuses placed in their home pens), considering that four of the present cows pushed the maximum weight of 178 kg (around 23% of the average bodyweight) in a relatively short period, it is likely that they may have pushed more if it had been made possible. Hence, had it been possible to place more weight on the gates, we may had observed an even higher maximum weight pushed. Yet, it is important to state that the maximum price does not reflect the cows’ ability to push the gate but the cows’ motivation to push for a given reward. Additionally, the censored latency to feed essentially reflects the motivation to access the feed reward. However, we cannot exclude other influences, such as some degree of fearfulness or boldness, which may have contributed to the variations in latencies to feed. Furthermore, the present findings need careful interpretation when compared with other studies using the same motivation test paradigm. Different food types, differences in reward duration and access to feed outside the test situation may affect an animal’s motivation to perform a certain behaviour^23^ and complicates comparisons of maximum price between experiments. Motivation tests outside the home environment have been criticised for leaving the animals no alternative to working for the resource^23^. Therefore, cost-free barley straw was offered as an alternative food to assure that the cows had something to feed on when they were not motivated to push the required weight for concentrate. Barley straw was chosen because it represented a familiar feed with very little nutritional value.

In intensive animal production, the practice of restricting energy intake, quantitatively or qualitatively, is not exclusive to dairy cows around dry-off and during the dry period. Pregnant sows^49,50^ and broiler breeders^51,52^ are also subjected to restricted diets to obtain ideal productivity and avoid later health and reproductive problems. Previous studies have demonstrated that both species undergo hunger due to insufficient energy intake (e.g. broiler breeders^53^ and pregnant sows^54^). Similarly, our study demonstrated that the provision of a low energy feed during 1 week before the last milking caused hunger in the experimental cows. On one hand, downgrading the diet quality can be stressful^4,13^ and detrimental to the welfare of cows. On the other hand, it reduces milk yield before the last milking^2,13^, and the cow avoids an abrupt dietary change on the last day of milking. Although we found no effect on feeding motivation of reduced daily milking frequency, this may reduce milk synthesis prior to the onset of the dry period^5^ and lower the incidence of milk leakage and intramammary infections in the dry period^12,15^. However, in the short term, less-frequent milking can lead to milk accumulation in the udder, consequent udder engorgement and increased udder pressure that may result in discomfort^11^. Aiming to avoid the problems associated with drying-off, recent studies investigated the effects of a short or omitted dry period on the health and behaviour of dairy cows^7,55,56^. Overall, these studies claim that extending the lactation period, or even omitting the dry period, leads to improved energy balance in cows during early lactation, besides avoiding dietary changes and regrouping associated with calving^10,55^. Nonetheless, cows with no dry-period lay down less during the weeks before calving^55^, and they were not separated from the resident group until the moment of calving, which has previously been found to prolong the calving process^57^. In addition, the renewal of udder cells may be compromised, causing a lower milk yield in the next lactation^7^, and a short or absent dry period may have negative effects on the health^56^.

In summary, this study sheds light on the importance of reviewing conventional dry-off protocols to improve the welfare of high-yielding cows. Further research is necessary to understand all potential impacts of drying-off on the welfare of dairy cows and tailor dry-off management that take the welfare of the cow, as well as the requirements of the farmer, into consideration. More specifically, future studies should investigate how a potential state of hunger induced by drying-off can affect the health (e.g. susceptibility to health disorders), behaviour (e.g. feeding patterns) and emotional experiences (e.g. cognitive bias) of dairy cows.

## Conclusions

The results of the current study show that management including a substantial reduction in energy intake to reduce milk synthesis in high-yielding cows prior to dry-off, despite the *ad libitum* availability of the feed, causes an increased feeding motivation which we interpret as hunger. The two measures, latency to feed and maximum price paid, were obtained by use of an operant push-gate methodology and confirmed that cows on an energy-reduced diet were more motivated to feed, and hence hungrier, than cows receiving a normal lactation diet. Yet, the ceiling of the maximum price might have underestimated cows’ feeding motivation. Furthermore, future feeding motivation studies should ponder the choice of feed reward that is attractive and does not jeopardise the closed economy of the test, the experimental design or the health of the experimental animals. Finally, this research shows how developing a methodology that encourages behaviours related to the target motivation can be used to evaluate the consequences of feeding management on animal welfare.

## Supplementary information

Supplementary information

Supplementary information: Cow pushing the gate and feeding

Supplementary data

## Data Availability

Raw data is available in the Supplementary Material. The data include the training phase, testing, characteristics of the cows, calibration of the gates, daily feed intake, average milk yield and energy intake during the 7-day experimental period.
