# Supplementary material for: Dairy cows fed a low energy diet before dry-off show signs of hunger despite ad libitum access
Source: Sci Rep. 2019 Nov 6;9:16159. doi: 10.1038/s41598-019-51866-7 (PMC6834606; doi:10.1038/s41598-019-51866-7)
Supplement: Supplementary file 1 — Supplementary information [file 41598_2019_51866_MOESM1_ESM.docx]

Dairy cows fed a low energy diet before dry-off show signs of hunger despite *ad libitum* access

Guilherme Amorim Franchi, Mette S. Herskin and Margit Bak Jensen

Animals and housing in the resident herd

The resident herd consisted of two groups of approximately 60 cows (primiparous and multiparous), each housed in adjacent mirror-image free-stall pens with concrete slatted floor in a naturally ventilated barn. The pens featured a minimum of one cubicle per cow (1.85 x 1.20 m, length to brisket board), and these were equipped with 70 mm mattresses (Cowtex, Tromborg staldudstyr og -inventar, Varde, Denmark) covered by a thin layer of sawdust, which was topped automatically (JHminiStrø, JH Staldservice A/S, Holstebro, Denmark) twice daily. Cows had access to water for ad libitum intake from a total of four 126 x 60 cm (length x width) self-filling water troughs, and a mechanical rotating cow brush (DeLaval, Tumba, Sweden) per group and were milked by an automatic milking system (AMS) (DeLaval, Tumba, Sweden). From 150 days in milk (DIM), cow were allowed to be milked at an interval of minimum 8 h (7 h for first lactation cows) or 9 kg of milk per milking. The milking interval of individual cows was based on information from previous milkings and DIM. The lactating cows were fed a standard partially mixed ration (PMR) for ad libitum intake from computerised feed bins (Insentec B.V., Marknesse, The Netherlands) and allowed to feed 3 kg of concentrate daily in the AMS. In the resident herd, 2-3 cows shared a particular feed bin.

Composition of the diets

Table x. Composition of diets (g/kg DM unless otherwise noted)

|  | Lactation cow rations | | Dry cow ration |
| --- | --- | --- | --- |
|  | Normal | Energy-reduced |  |
| Compound feed, pelleted^a^ | 131 | 110 |  |
| Maize silage | 276 | 169 | 524 |
| Grass-clover silage | 233 | 142 | 157.1 |
| Straw, barley |  | 294 | 227 |
| Barley, rolled | 126 | 77.1 |  |
| Rape seed cakes | 77 | 47.2 | 35 |
| Soybean meal | 77 | 47.2 | 35 |
| Sugar beet pulp, dried | 68 | 42 |  |
| Calcium carbonate | 0.8 | 6.6 | 4.4 |
| Monocalcium phosphate |  | 30.6 | 4.4 |
| Magnesium oxide |  | 6.1 |  |
| Sodium chloride | 1.0 | 6.7 | 3.5 |
| Sodium bicarbonate | 2.9 | 7.9 |  |
| Premix | 5.0^b^ | 12.2^b^ | 9.6^c^ |
| Urea | 2.3 | 1.4 |  |
| Forage:Concentrate ratio | 51:49 | 65:35 | 92:08 |
| Nutrients^d^ |  |  |  |
| Net energy for lactation, MJ/kg DM | 6.75 | 5.73 | 5.71 |
| Crude protein | 166 | 125 | 92 |
| Ca, g/d | 125 | 129 | 64 |
| P, g/d | 78 | 81 | 42 |
| Na, g/d | 47 | 49 | 25 |
| Mg, g/d | 49 | 52 | 36 |
| K, g/d | 283 | 129 | 171 |
| Dietary cation-anion difference, meq/kg DM | 224 | 220 | 121 |

^a^Composed of (g/kg basis): 170 dried sugar beet pulp, 168 rape seed meal, 146 barley, 146 wheat, 90 soybean meal, 70 dried citrus pulp, 70 sunflower meal, 50 dried grass meal, 50 wheat bran, 22 sugar beet molasses, 10 minerals and vitamins, 8 palm oil.

^b^Premix lactation.

^c^Premix dry cow

^d^Calculated according to NorFor^1^.

Animals and housing during the experimental period

During the experimental period (D-7 to D+7 relative to the last milking on the dry-off day), enrolled cows were kept in an experimental pen in the same barn as the resident pens consisting of an alley (8 m x 2.6 m) with 10 adjacent cubicles (six measuring 1.4 m x 1.8 m and four measuring 1.3 m x 1.8 m) and a feeding area (8.6 m x 4 m). The alley and the feeding area were lined with concrete slatted flooring (slats = 15 cm, spacing = 4 cm), and the cubicles were lined with mattresses (Cowtex, Tromborg staldudstyr og -inventar, Varde, Denmark) automatically topped with sawdust (JH-miniStrø, JH Staldservice A/S, Holstebro, Denmark) twice a day. The cubicles were scraped manually twice daily and the alleys were scraped continuously by automatic cleaning robots (Lely Discovery, Lely Holding, Maassluis, The Netherlands). The feeding area was equipped with computerised feed bins (1 bin/cow) (Insentec BV, Marknesse, The Netherlands), a mechanical rotating cow brush (DeLaval, Tumba, Sweden) and a 126 x 60 cm self-filling water trough. The cows were taken to be milked in a standard AMS (DeLaval AB, Tumba, Sweden) via a corridor operated by farm staff. The number of cows per 2-week batch varied from 1-6 depending on their availability. If there was only one cow in a batch, a non-experimental companion cow was added to the pen.

Illustrations


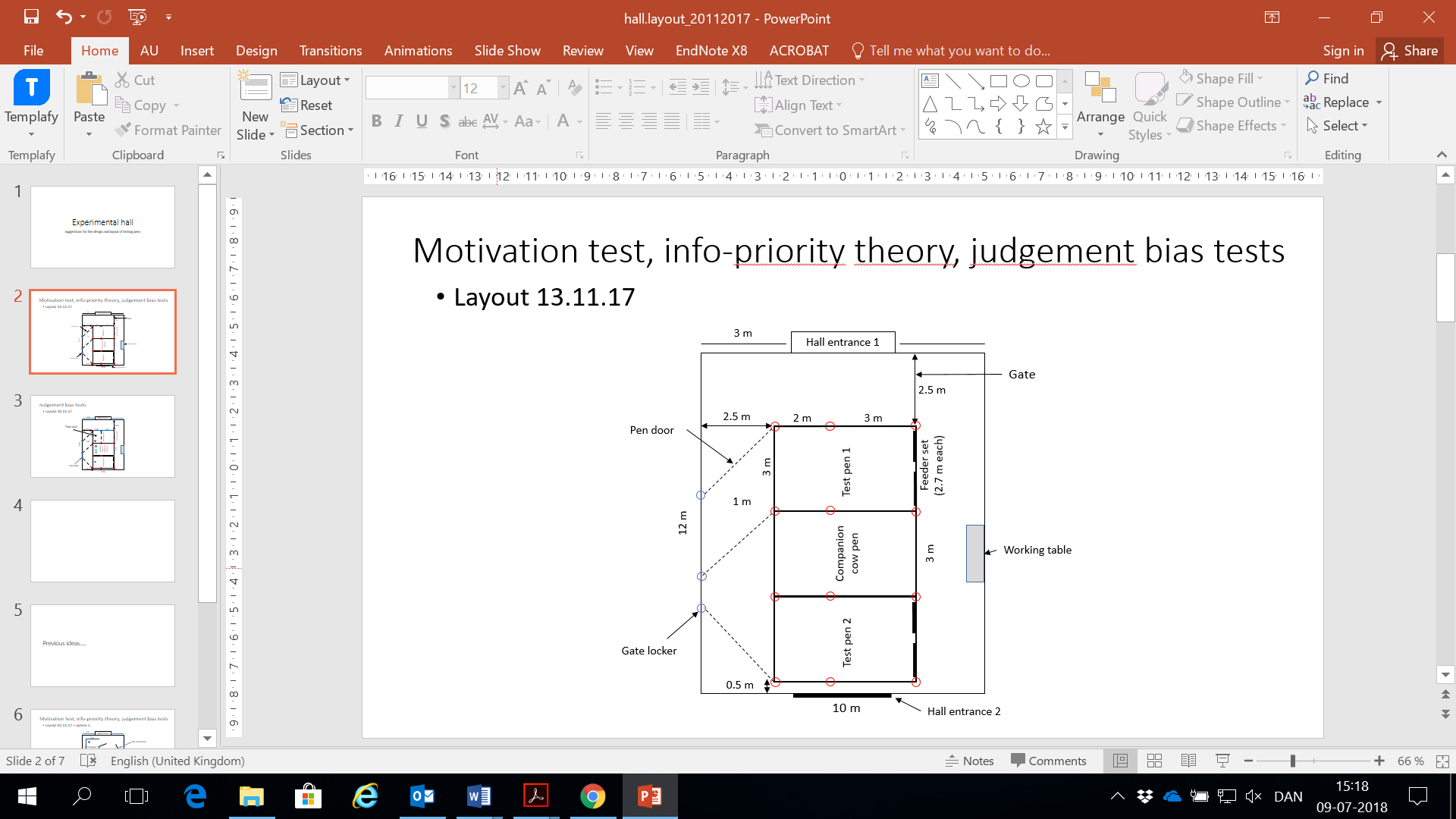


Supplementary figure 1. Layout of the experimental hall used for push-gate testing of the dairy cows. Cows entered the hall through the entrance 1.


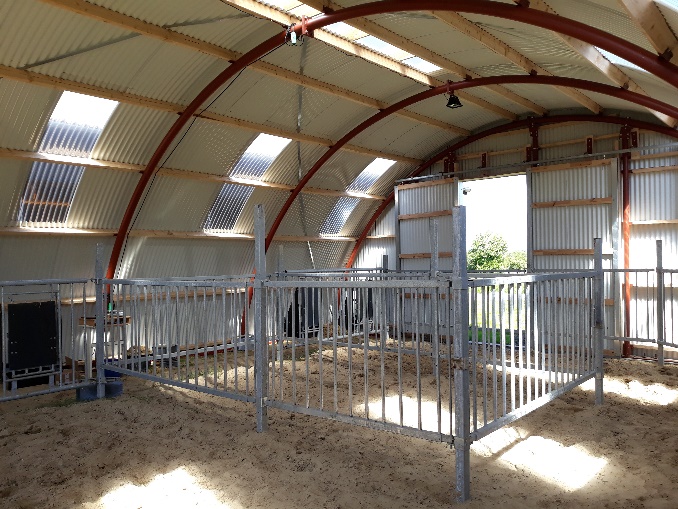


Supplementary figure 2. Internal view of the experimental hall. This picture taken from the entrance illustrates the two pens equipped with the push-gate feeders and the companion cow pen in between.


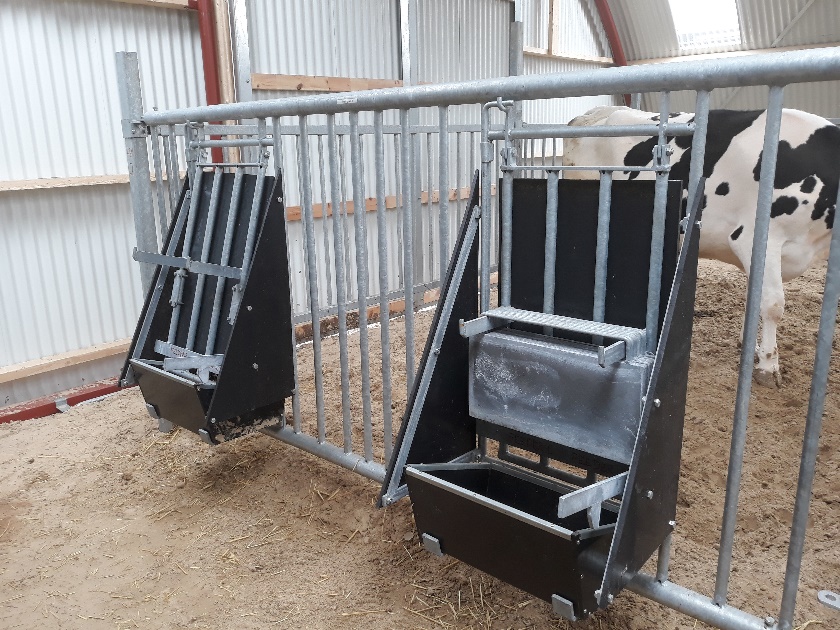


Supplementary figure 3. Push-gate feeders installed in the test pens. The push-gate feeder equipped with 10-kg metal plates was the one receiving concentrate (feed reward). The other push-gate feeder contained no-cost barley straw and was permanently open during testing.

Reference:

1. Volden, H. NorFor—The Nordic Feed Evaluation System. EAAP publication no. 130. Wageningen Academic Publishers, Wageningen, the Netherlands (2011).
